# 6a-Nitro-6-phenyl-6,6a,6b,7,8,9,10,12a-octa­hydro­spiro­[chromeno[3,4-*a*]indol­izine-12,3′-indolin]-2′-one

**DOI:** 10.1107/S1600536813014062

**Published:** 2013-05-31

**Authors:** Seenivasan Karthiga Devi, Thothadri Srinivasan, Jonnalagadda Naga Siva Rao, Raghavachary Raghunathan, Devadasan Velmurugan

**Affiliations:** a Centre of Advanced Study in Crystallography and Biophysics, University of Madras, Guindy Campus, Chennai 600 025, India; b Department of Organic Chemistry, University of Madras, Guindy Campus, Chennai 600 025, India

## Abstract

In the title compound, C_28_H_25_N_3_O_4_, the central pyrrolidine ring adopts adopts an envelope conformation with the N atom as the flap and the piperidine ring adopts a chair conformation. The pendant pyrrolidine ring is almost planar (r.m.s. deviation = 0.008 Å). An intra­molecular C—H⋯O inter­action closes an *S*(6) ring. In the crystal, inversion dimers linked by pairs of N—H⋯O hydrogen bonds generate *R*_2_^2^(8) loops.

## Related literature

For biological background to 4*H*-chromene derivatives, see: Cai (2008 ▶); Valenti *et al.* (1993 ▶). For applications of indoline-2-one and its derivatives as precursors in the synthesis of pharmaceuticals, see: Colgan *et al.* (1996 ▶).
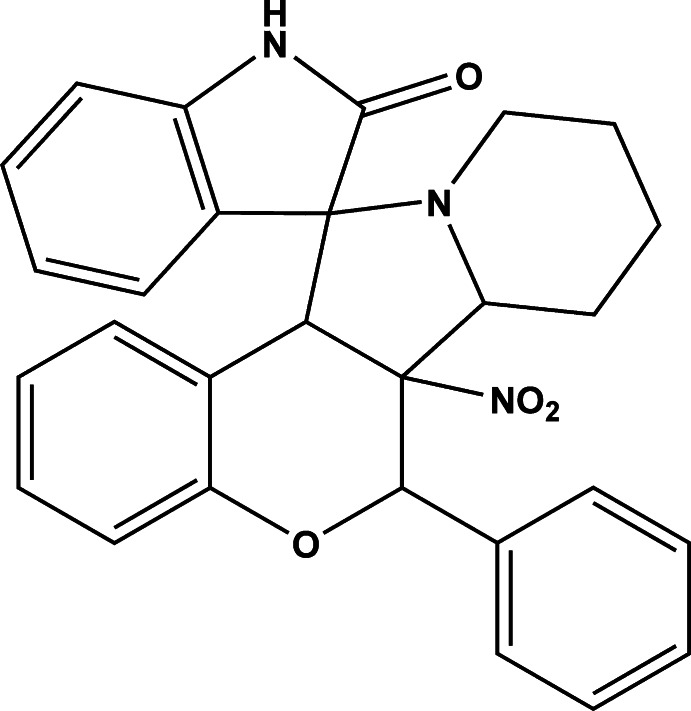


## Experimental

### 

#### Crystal data

C_28_H_25_N_3_O_4_
*M* *_r_* = 467.51
Monoclinic, 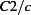

*a* = 23.2940 (13) Å
*b* = 11.2517 (7) Å
*c* = 19.7702 (10) Å
β = 112.659 (3)°
*V* = 4781.8 (5) Å^3^
*Z* = 8
Mo *K*α radiation
μ = 0.09 mm^−1^
*T* = 293 K
0.30 × 0.25 × 0.20 mm

#### Data collection

Bruker SMART APEXII CCD diffractometer
Absorption correction: multi-scan (*SADABS*; Bruker, 2008 ▶) *T*_min_ = 0.974, *T*_max_ = 0.983
20735 measured reflections
5804 independent reflections
3892 reflections with *I* > 2σ(*I*)
*R*_int_ = 0.048

#### Refinement

*R*[*F* ^2^ > 2σ(*F* ^2^)] = 0.046
*wR*(*F* ^2^) = 0.127
*S* = 1.00
5804 reflections
316 parameters
H-atom parameters constrained
Δρ_max_ = 0.22 e Å^−3^
Δρ_min_ = −0.22 e Å^−3^

### 

Data collection: *APEX2* (Bruker, 2008 ▶); cell refinement: *SAINT* (Bruker, 2008 ▶); data reduction: *SAINT*; program(s) used to solve structure: *SHELXS97* (Sheldrick, 2008 ▶); program(s) used to refine structure: *SHELXL97* (Sheldrick, 2008 ▶); molecular graphics: *ORTEP-3 for Windows* (Farrugia, 2012 ▶); software used to prepare material for publication: *SHELXL97* and *PLATON* (Spek, 2009 ▶).

## Supplementary Material

Crystal structure: contains datablock(s) global, I. DOI: 10.1107/S1600536813014062/hb7084sup1.cif

Structure factors: contains datablock(s) I. DOI: 10.1107/S1600536813014062/hb7084Isup2.hkl

Supplementary material file. DOI: 10.1107/S1600536813014062/hb7084Isup3.cml

Additional supplementary materials:  crystallographic information; 3D view; checkCIF report

## Figures and Tables

**Table 1:** Hydrogen-bond geometry (Å, °)

*D*—H⋯*A*	*D*—H	H⋯*A*	*D*⋯*A*	*D*—H⋯*A*
C16—H16⋯O4	0.98	2.50	3.1394 (18)	123
N3—H3*A*⋯O4^i^	0.86	1.97	2.8218 (17)	168

Symmetry code: (i) 
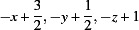
.

**Fig. 1.**
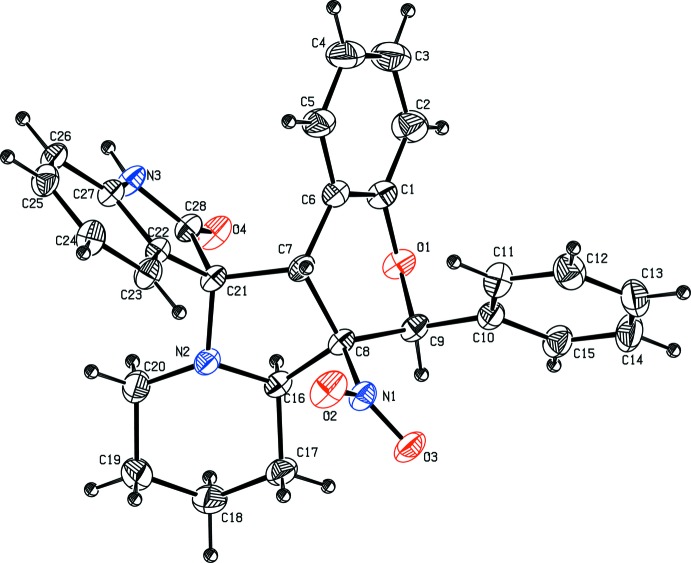
The molecular structure of the title compound, showing displacement ellipsoids drawn at the 30% probability level.

**Fig. 2.**
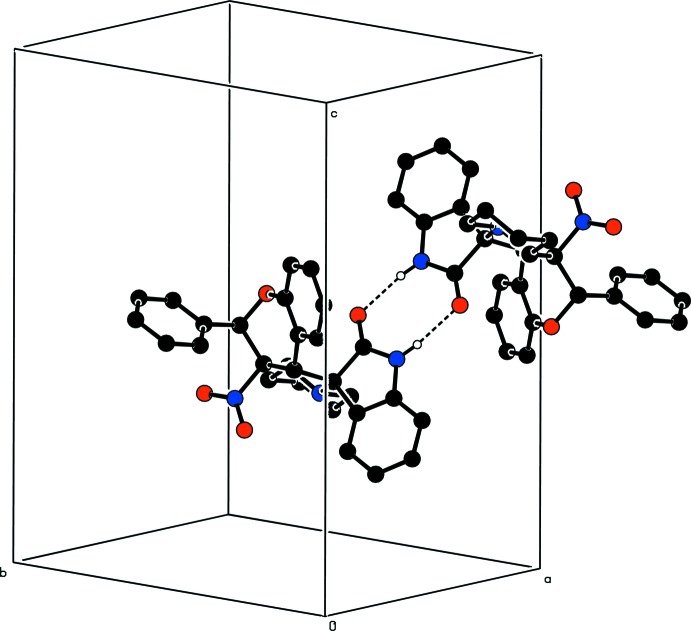
An inversion dimer in the crystal of the title compound viewed down [100].

## supplementary crystallographic information

### Comment

4H-Chromenes are biologically important compounds used in the drug-discovery process (e.g. Valenti *et al.*, 1993; Cai, 2008). Indoline-2-one and its derivatives have been used as precursors to synthesis pharmaceuticals (Colgan *et al.*, 1996). Continuing our interest in such compounds we have synthesized the title compound and report herein its crystal structure.

In the title compound, C_28_H_25_N_3_O_4_, (fig.1) the pyrrolidine ring adopts an envelope conformation and the piperidine ring adopts a *chair* conformation. The pyrrolidine ring (N2/C7/C8/C16/C21) makes a dihedral angle of 84.13 (9)° with the other pyrrolidine ring (N3/C21/C22/C27/C28) which shows that they are almost orthogonal to each other. The pyrrolidine ring makes a dihedral angle of 61.22 (8)° with the the pyran ring (O1/C1/C6-C9), it makes a dihedral angle of 4.00 (9)° with the piperidine ring (N2/C16-C20).

The other pyrrolidine ring (N3/C21/C22/C27/C28) makes a dihedral angle of 39.60 (9)° with the pyran ring, it makes a dihedral angle of 88.12 (9)° with the piperidine ring which shows that they are almost at right angles to each other. The dihedral angle between the pyran ring and the piperidine ring is 57.58 (9)° . The oxygen atom O4 attached with pyrrolidine ring deviates by -0.0736 (1)Å. The nitrogroup attached with the pyrrolidine ring makes a diherdal angle of 88.81 (2)° which shows it is in orthogonal orientation . The crystal packing features N—H···O hydrogen bonds and an intramolecular C—H···O hydrogen bond is also observed.

### Experimental

To a solution of isatin (1 equiv) and piperidine-2-carboxylic acid (1.4 equiv) in dry toluene, was added 3-nitro-2-phenyl-2H-chromene (1equiv) under nitrogen atmosphere. The reaction mixture was refluxed f or 24h in Dean-Stark apparatus to give the cycloadducts. After completion of the reaction as indicated by TLC, the solvent was evaporated under reduced pressure. The crude product was extracted with dichloromethane. The organic layer was dried with anhydrous sodium sulphate and concentrated in vacuo. Then the crude product was purified by column chromatography using hexane/EtOAc (7:3) as eluent. Colourless blocks were obtained by slow evaporation of a solution of the title compound in ethyl acetate at room temperature.

### Refinement

The hydrogen atoms were placed in calculated positions and treated as riding atoms: C—H = 0.93 Å to 0.97 Å, and N—H =0.86 Å with Uiso(H) = 1.5Ueq(C) for methyl H atoms and = 1.2Ueq(C) for other H atoms.

### Crystal data

**Table:**

C_28_H_25_N_3_O_4_	*F*(000) = 1968
*M**_r_* = 467.51	*D*_x_ = 1.299 Mg m^−^^3^
Monoclinic, *C*2/*c*	Mo *K*α radiation, λ = 0.71073 Å
Hall symbol: -C 2yc	Cell parameters from 5804 reflections
*a* = 23.2940 (13) Å	θ = 1.9–28.3°
*b* = 11.2517 (7) Å	µ = 0.09 mm^−^^1^
*c* = 19.7702 (10) Å	*T* = 293 K
β = 112.659 (3)°	Block, colourless
*V* = 4781.8 (5) Å^3^	0.30 × 0.25 × 0.20 mm
*Z* = 8	

### Data collection

**Table:**

Bruker SMART APEXII CCD diffractometer	5804 independent reflections
Radiation source: fine-focus sealed tube	3892 reflections with *I* > 2σ(*I*)
Graphite monochromator	*R*_int_ = 0.048
ω and φ scans	θ_max_ = 28.3°, θ_min_ = 1.9°
Absorption correction: multi-scan (*SADABS*; Bruker, 2008)	*h* = −30→27
*T*_min_ = 0.974, *T*_max_ = 0.983	*k* = −14→14
20735 measured reflections	*l* = −25→25

### Refinement

**Table:**

Refinement on *F*^2^	Primary atom site location: structure-invariant direct methods
Least-squares matrix: full	Secondary atom site location: difference Fourier map
*R*[*F*^2^ > 2σ(*F*^2^)] = 0.046	Hydrogen site location: inferred from neighbouring sites
*wR*(*F*^2^) = 0.127	H-atom parameters constrained
*S* = 1.00	*w* = 1/[σ^2^(*F*_o_^2^) + (0.0505*P*)^2^ + 2.441*P*] where *P* = (*F*_o_^2^ + 2*F*_c_^2^)/3
5804 reflections	(Δ/σ)_max_ < 0.001
316 parameters	Δρ_max_ = 0.22 e Å^−^^3^
0 restraints	Δρ_min_ = −0.22 e Å^−^^3^

### Special details

**Table:**

Geometry. All esds (except the esd in the dihedral angle between two l.s. planes) are estimated using the full covariance matrix. The cell esds are taken into account individually in the estimation of esds in distances, angles and torsion angles; correlations between esds in cell parameters are only used when they are defined by crystal symmetry. An approximate (isotropic) treatment of cell esds is used for estimating esds involving l.s. planes.
Refinement. Refinement of F^2^ against ALL reflections. The weighted R-factor wR and goodness of fit S are based on F^2^, conventional R-factors R are based on F, with F set to zero for negative F^2^. The threshold expression of F^2^ > 2sigma(F^2^) is used only for calculating R-factors(gt) etc. and is not relevant to the choice of reflections for refinement. R-factors based on F^2^ are statistically about twice as large as those based on F, and R- factors based on ALL data will be even larger.

### Fractional atomic coordinates and isotropic or equivalent isotropic displacement parameters (Å^2^)

**Table:**

	*x*	*y*	*z*	*U*_iso_*/*U*_eq_	
C1	0.85609 (7)	−0.07457 (14)	0.50076 (8)	0.0424 (4)	
C2	0.86024 (10)	−0.05264 (18)	0.43391 (10)	0.0608 (5)	
H2	0.8376	−0.0985	0.3932	0.073*	
C3	0.89794 (11)	0.0370 (2)	0.42802 (12)	0.0740 (6)	
H3	0.8998	0.0536	0.3828	0.089*	
C4	0.93302 (11)	0.1027 (2)	0.48873 (12)	0.0725 (6)	
H4	0.9592	0.1624	0.4848	0.087*	
C5	0.92917 (8)	0.07964 (16)	0.55536 (10)	0.0530 (4)	
H5	0.9531	0.1237	0.5963	0.064*	
C6	0.89013 (7)	−0.00828 (13)	0.56210 (8)	0.0384 (3)	
C7	0.88197 (6)	−0.02739 (12)	0.63324 (7)	0.0333 (3)	
H7	0.9232	−0.0262	0.6731	0.040*	
C8	0.85040 (7)	−0.14626 (12)	0.63696 (7)	0.0337 (3)	
C9	0.83905 (7)	−0.23119 (13)	0.57169 (8)	0.0383 (3)	
H9	0.8051	−0.2843	0.5699	0.046*	
C10	0.89189 (7)	−0.30987 (14)	0.57303 (8)	0.0399 (4)	
C11	0.95412 (8)	−0.27872 (16)	0.60526 (10)	0.0514 (4)	
H11	0.9652	−0.2047	0.6274	0.062*	
C12	0.99985 (9)	−0.35599 (18)	0.60498 (10)	0.0592 (5)	
H12	1.0415	−0.3343	0.6271	0.071*	
C13	0.98385 (11)	−0.46543 (19)	0.57194 (10)	0.0629 (5)	
H13	1.0147	−0.5178	0.5718	0.075*	
C14	0.92229 (11)	−0.49735 (17)	0.53910 (11)	0.0660 (6)	
H14	0.9115	−0.5711	0.5165	0.079*	
C15	0.87660 (9)	−0.42018 (15)	0.53967 (9)	0.0505 (4)	
H15	0.8350	−0.4423	0.5174	0.061*	
C16	0.78884 (7)	−0.11043 (13)	0.64328 (8)	0.0364 (3)	
H16	0.7581	−0.0947	0.5938	0.044*	
C17	0.75963 (8)	−0.19292 (15)	0.68128 (10)	0.0490 (4)	
H17A	0.7892	−0.2104	0.7304	0.059*	
H17B	0.7481	−0.2671	0.6544	0.059*	
C18	0.70199 (9)	−0.13326 (17)	0.68491 (11)	0.0585 (5)	
H18A	0.6715	−0.1204	0.6357	0.070*	
H18B	0.6836	−0.1846	0.7105	0.070*	
C19	0.71962 (9)	−0.01512 (18)	0.72468 (12)	0.0624 (5)	
H19A	0.6825	0.0240	0.7248	0.075*	
H19B	0.7474	−0.0288	0.7752	0.075*	
C20	0.75132 (8)	0.06411 (15)	0.68767 (11)	0.0535 (4)	
H20A	0.7221	0.0850	0.6390	0.064*	
H20B	0.7650	0.1369	0.7156	0.064*	
C21	0.84106 (6)	0.07028 (12)	0.64953 (7)	0.0335 (3)	
C22	0.87723 (7)	0.17155 (12)	0.69677 (8)	0.0339 (3)	
C23	0.92160 (7)	0.17261 (14)	0.76717 (8)	0.0420 (4)	
H23	0.9341	0.1023	0.7935	0.050*	
C24	0.94731 (8)	0.28065 (15)	0.79802 (9)	0.0476 (4)	
H24	0.9772	0.2829	0.8456	0.057*	
C25	0.92886 (8)	0.38464 (15)	0.75866 (9)	0.0460 (4)	
H25	0.9469	0.4561	0.7801	0.055*	
C26	0.88421 (7)	0.38502 (14)	0.68819 (9)	0.0422 (4)	
H26	0.8716	0.4554	0.6619	0.051*	
C27	0.85910 (7)	0.27729 (13)	0.65836 (8)	0.0359 (3)	
C28	0.79893 (7)	0.13765 (13)	0.57914 (8)	0.0372 (3)	
N1	0.88825 (6)	−0.21232 (12)	0.70783 (7)	0.0411 (3)	
N2	0.80488 (6)	0.00268 (11)	0.68254 (7)	0.0375 (3)	
N3	0.81272 (6)	0.25411 (11)	0.58899 (7)	0.0435 (3)	
H3A	0.7953	0.3079	0.5567	0.052*	
O1	0.81579 (5)	−0.16138 (10)	0.50491 (5)	0.0439 (3)	
O2	0.92442 (6)	−0.15660 (11)	0.75982 (6)	0.0606 (4)	
O3	0.87676 (6)	−0.31771 (10)	0.70992 (6)	0.0546 (3)	
O4	0.75833 (5)	0.09239 (9)	0.52555 (6)	0.0476 (3)	

### Atomic displacement parameters (Å^2^)

**Table:**

	*U*^11^	*U*^22^	*U*^33^	*U*^12^	*U*^13^	*U*^23^
C1	0.0445 (9)	0.0376 (9)	0.0414 (8)	−0.0021 (7)	0.0124 (7)	0.0040 (7)
C2	0.0756 (13)	0.0620 (12)	0.0431 (9)	−0.0079 (10)	0.0209 (9)	−0.0001 (9)
C3	0.1009 (17)	0.0740 (14)	0.0602 (12)	−0.0132 (13)	0.0456 (12)	0.0058 (11)
C4	0.0894 (15)	0.0648 (14)	0.0804 (14)	−0.0232 (12)	0.0516 (13)	0.0011 (11)
C5	0.0565 (11)	0.0469 (10)	0.0600 (10)	−0.0152 (8)	0.0272 (9)	−0.0037 (8)
C6	0.0386 (8)	0.0327 (8)	0.0425 (8)	−0.0006 (6)	0.0141 (7)	0.0027 (6)
C7	0.0309 (7)	0.0269 (7)	0.0351 (7)	−0.0028 (6)	0.0050 (6)	0.0011 (6)
C8	0.0346 (7)	0.0267 (7)	0.0340 (7)	−0.0007 (6)	0.0067 (6)	0.0034 (6)
C9	0.0416 (8)	0.0291 (7)	0.0381 (8)	−0.0055 (6)	0.0085 (7)	0.0004 (6)
C10	0.0484 (9)	0.0334 (8)	0.0350 (7)	−0.0005 (7)	0.0130 (7)	−0.0001 (6)
C11	0.0492 (10)	0.0438 (10)	0.0557 (10)	0.0023 (8)	0.0142 (8)	−0.0056 (8)
C12	0.0548 (11)	0.0661 (13)	0.0552 (10)	0.0120 (10)	0.0195 (9)	0.0024 (9)
C13	0.0809 (14)	0.0625 (13)	0.0500 (10)	0.0290 (11)	0.0305 (10)	0.0065 (9)
C14	0.1004 (17)	0.0413 (10)	0.0585 (11)	0.0087 (11)	0.0328 (12)	−0.0093 (9)
C15	0.0646 (11)	0.0389 (9)	0.0447 (9)	−0.0042 (8)	0.0175 (8)	−0.0054 (7)
C16	0.0354 (8)	0.0300 (8)	0.0397 (8)	−0.0029 (6)	0.0101 (6)	0.0021 (6)
C17	0.0516 (10)	0.0371 (9)	0.0606 (10)	−0.0074 (8)	0.0241 (9)	0.0033 (8)
C18	0.0541 (11)	0.0533 (11)	0.0776 (13)	−0.0084 (9)	0.0360 (10)	0.0040 (10)
C19	0.0634 (12)	0.0560 (12)	0.0832 (13)	−0.0019 (10)	0.0452 (11)	−0.0041 (10)
C20	0.0532 (10)	0.0387 (10)	0.0753 (12)	0.0009 (8)	0.0319 (9)	−0.0037 (8)
C21	0.0341 (7)	0.0262 (7)	0.0345 (7)	−0.0002 (6)	0.0068 (6)	0.0029 (6)
C22	0.0345 (7)	0.0273 (7)	0.0379 (7)	−0.0009 (6)	0.0117 (6)	−0.0001 (6)
C23	0.0423 (9)	0.0351 (8)	0.0399 (8)	0.0034 (7)	0.0063 (7)	0.0011 (6)
C24	0.0427 (9)	0.0447 (10)	0.0441 (9)	0.0005 (8)	0.0041 (7)	−0.0085 (7)
C25	0.0460 (9)	0.0358 (9)	0.0540 (9)	−0.0071 (7)	0.0167 (8)	−0.0114 (7)
C26	0.0504 (9)	0.0266 (8)	0.0491 (9)	−0.0019 (7)	0.0186 (8)	0.0004 (6)
C27	0.0371 (8)	0.0299 (8)	0.0377 (7)	−0.0014 (6)	0.0110 (6)	0.0011 (6)
C28	0.0361 (8)	0.0306 (8)	0.0394 (8)	0.0009 (6)	0.0085 (6)	0.0033 (6)
N1	0.0458 (7)	0.0324 (7)	0.0415 (7)	0.0043 (6)	0.0126 (6)	0.0043 (5)
N2	0.0387 (7)	0.0285 (6)	0.0452 (7)	−0.0016 (5)	0.0159 (6)	0.0010 (5)
N3	0.0499 (8)	0.0264 (7)	0.0408 (7)	0.0003 (6)	0.0026 (6)	0.0066 (5)
O1	0.0446 (6)	0.0399 (6)	0.0360 (5)	−0.0074 (5)	0.0030 (5)	0.0022 (4)
O2	0.0669 (8)	0.0510 (7)	0.0413 (6)	−0.0021 (6)	−0.0040 (6)	0.0037 (6)
O3	0.0733 (8)	0.0299 (6)	0.0580 (7)	0.0041 (6)	0.0225 (6)	0.0117 (5)
O4	0.0451 (6)	0.0350 (6)	0.0431 (6)	−0.0030 (5)	−0.0045 (5)	0.0032 (5)

### Geometric parameters (Å, º)

**Table:**

C1—O1	1.3790 (19)	C16—C17	1.511 (2)
C1—C6	1.384 (2)	C16—H16	0.9800
C1—C2	1.384 (2)	C17—C18	1.527 (2)
C2—C3	1.372 (3)	C17—H17A	0.9700
C2—H2	0.9300	C17—H17B	0.9700
C3—C4	1.378 (3)	C18—C19	1.518 (3)
C3—H3	0.9300	C18—H18A	0.9700
C4—C5	1.379 (3)	C18—H18B	0.9700
C4—H4	0.9300	C19—C20	1.514 (3)
C5—C6	1.385 (2)	C19—H19A	0.9700
C5—H5	0.9300	C19—H19B	0.9700
C6—C7	1.505 (2)	C20—N2	1.464 (2)
C7—C8	1.5417 (19)	C20—H20A	0.9700
C7—C21	1.567 (2)	C20—H20B	0.9700
C7—H7	0.9800	C21—N2	1.4624 (18)
C8—N1	1.5301 (18)	C21—C22	1.5080 (19)
C8—C16	1.540 (2)	C21—C28	1.5563 (19)
C8—C9	1.544 (2)	C22—C23	1.377 (2)
C9—O1	1.4499 (17)	C22—C27	1.387 (2)
C9—C10	1.508 (2)	C23—C24	1.388 (2)
C9—H9	0.9800	C23—H23	0.9300
C10—C11	1.385 (2)	C24—C25	1.379 (2)
C10—C15	1.386 (2)	C24—H24	0.9300
C11—C12	1.377 (2)	C25—C26	1.381 (2)
C11—H11	0.9300	C25—H25	0.9300
C12—C13	1.376 (3)	C26—C27	1.376 (2)
C12—H12	0.9300	C26—H26	0.9300
C13—C14	1.375 (3)	C27—N3	1.4050 (19)
C13—H13	0.9300	C28—O4	1.2264 (17)
C14—C15	1.377 (3)	C28—N3	1.3454 (19)
C14—H14	0.9300	N1—O3	1.2199 (17)
C15—H15	0.9300	N1—O2	1.2216 (17)
C16—N2	1.4623 (18)	N3—H3A	0.8600
			
O1—C1—C6	120.19 (14)	C16—C17—H17A	109.9
O1—C1—C2	118.86 (15)	C18—C17—H17A	109.9
C6—C1—C2	120.91 (16)	C16—C17—H17B	109.9
C3—C2—C1	119.64 (18)	C18—C17—H17B	109.9
C3—C2—H2	120.2	H17A—C17—H17B	108.3
C1—C2—H2	120.2	C19—C18—C17	109.99 (15)
C2—C3—C4	120.33 (18)	C19—C18—H18A	109.7
C2—C3—H3	119.8	C17—C18—H18A	109.7
C4—C3—H3	119.8	C19—C18—H18B	109.7
C3—C4—C5	119.73 (18)	C17—C18—H18B	109.7
C3—C4—H4	120.1	H18A—C18—H18B	108.2
C5—C4—H4	120.1	C20—C19—C18	110.49 (15)
C4—C5—C6	120.89 (17)	C20—C19—H19A	109.6
C4—C5—H5	119.6	C18—C19—H19A	109.6
C6—C5—H5	119.6	C20—C19—H19B	109.6
C1—C6—C5	118.47 (15)	C18—C19—H19B	109.6
C1—C6—C7	120.39 (13)	H19A—C19—H19B	108.1
C5—C6—C7	121.06 (14)	N2—C20—C19	110.06 (14)
C6—C7—C8	113.75 (12)	N2—C20—H20A	109.6
C6—C7—C21	113.41 (11)	C19—C20—H20A	109.6
C8—C7—C21	105.04 (11)	N2—C20—H20B	109.6
C6—C7—H7	108.1	C19—C20—H20B	109.6
C8—C7—H7	108.1	H20A—C20—H20B	108.2
C21—C7—H7	108.1	N2—C21—C22	113.40 (12)
N1—C8—C16	106.20 (11)	N2—C21—C28	112.27 (11)
N1—C8—C7	110.35 (11)	C22—C21—C28	101.25 (11)
C16—C8—C7	104.66 (11)	N2—C21—C7	103.15 (11)
N1—C8—C9	108.50 (11)	C22—C21—C7	114.73 (11)
C16—C8—C9	111.39 (12)	C28—C21—C7	112.45 (11)
C7—C8—C9	115.35 (12)	C23—C22—C27	119.84 (13)
O1—C9—C10	110.48 (12)	C23—C22—C21	130.92 (13)
O1—C9—C8	108.02 (11)	C27—C22—C21	109.24 (12)
C10—C9—C8	118.21 (12)	C22—C23—C24	118.71 (14)
O1—C9—H9	106.5	C22—C23—H23	120.6
C10—C9—H9	106.5	C24—C23—H23	120.6
C8—C9—H9	106.5	C25—C24—C23	120.47 (14)
C11—C10—C15	118.49 (16)	C25—C24—H24	119.8
C11—C10—C9	124.19 (14)	C23—C24—H24	119.8
C15—C10—C9	117.32 (15)	C24—C25—C26	121.47 (15)
C12—C11—C10	120.83 (17)	C24—C25—H25	119.3
C12—C11—H11	119.6	C26—C25—H25	119.3
C10—C11—H11	119.6	C27—C26—C25	117.41 (14)
C13—C12—C11	119.90 (19)	C27—C26—H26	121.3
C13—C12—H12	120.0	C25—C26—H26	121.3
C11—C12—H12	120.0	C26—C27—C22	122.11 (14)
C14—C13—C12	120.03 (18)	C26—C27—N3	128.46 (14)
C14—C13—H13	120.0	C22—C27—N3	109.43 (12)
C12—C13—H13	120.0	O4—C28—N3	126.24 (14)
C13—C14—C15	120.03 (18)	O4—C28—C21	125.52 (13)
C13—C14—H14	120.0	N3—C28—C21	108.16 (12)
C15—C14—H14	120.0	O3—N1—O2	124.03 (13)
C14—C15—C10	120.72 (18)	O3—N1—C8	116.53 (12)
C14—C15—H15	119.6	O2—N1—C8	119.25 (12)
C10—C15—H15	119.6	C16—N2—C21	106.81 (11)
N2—C16—C17	110.04 (13)	C16—N2—C20	113.42 (12)
N2—C16—C8	102.43 (11)	C21—N2—C20	116.01 (12)
C17—C16—C8	119.42 (13)	C28—N3—C27	111.91 (12)
N2—C16—H16	108.1	C28—N3—H3A	124.0
C17—C16—H16	108.1	C27—N3—H3A	124.0
C8—C16—H16	108.1	C1—O1—C9	114.45 (11)
C16—C17—C18	109.01 (14)		
			
O1—C1—C2—C3	177.03 (18)	C8—C7—C21—C22	140.86 (12)
C6—C1—C2—C3	−0.9 (3)	C6—C7—C21—C28	20.67 (16)
C1—C2—C3—C4	2.0 (3)	C8—C7—C21—C28	−104.13 (13)
C2—C3—C4—C5	−1.3 (4)	N2—C21—C22—C23	57.8 (2)
C3—C4—C5—C6	−0.5 (3)	C28—C21—C22—C23	178.28 (16)
O1—C1—C6—C5	−178.74 (14)	C7—C21—C22—C23	−60.4 (2)
C2—C1—C6—C5	−0.9 (3)	N2—C21—C22—C27	−121.55 (13)
O1—C1—C6—C7	−2.0 (2)	C28—C21—C22—C27	−1.08 (15)
C2—C1—C6—C7	175.85 (16)	C7—C21—C22—C27	120.27 (13)
C4—C5—C6—C1	1.5 (3)	C27—C22—C23—C24	−0.1 (2)
C4—C5—C6—C7	−175.15 (18)	C21—C22—C23—C24	−179.42 (15)
C1—C6—C7—C8	17.7 (2)	C22—C23—C24—C25	−0.3 (3)
C5—C6—C7—C8	−165.71 (14)	C23—C24—C25—C26	0.6 (3)
C1—C6—C7—C21	−102.30 (16)	C24—C25—C26—C27	−0.5 (2)
C5—C6—C7—C21	74.33 (18)	C25—C26—C27—C22	0.1 (2)
C6—C7—C8—N1	129.79 (13)	C25—C26—C27—N3	178.80 (15)
C21—C7—C8—N1	−105.62 (12)	C23—C22—C27—C26	0.2 (2)
C6—C7—C8—C16	−116.34 (13)	C21—C22—C27—C26	179.63 (14)
C21—C7—C8—C16	8.25 (13)	C23—C22—C27—N3	−178.70 (14)
C6—C7—C8—C9	6.41 (17)	C21—C22—C27—N3	0.74 (17)
C21—C7—C8—C9	131.00 (12)	N2—C21—C28—O4	−54.7 (2)
N1—C8—C9—O1	−167.40 (11)	C22—C21—C28—O4	−175.92 (15)
C16—C8—C9—O1	76.04 (14)	C7—C21—C28—O4	61.14 (19)
C7—C8—C9—O1	−43.05 (16)	N2—C21—C28—N3	122.35 (14)
N1—C8—C9—C10	−41.10 (17)	C22—C21—C28—N3	1.08 (16)
C16—C8—C9—C10	−157.65 (13)	C7—C21—C28—N3	−121.86 (14)
C7—C8—C9—C10	83.25 (16)	C16—C8—N1—O3	83.56 (15)
O1—C9—C10—C11	93.16 (18)	C7—C8—N1—O3	−163.55 (13)
C8—C9—C10—C11	−31.9 (2)	C9—C8—N1—O3	−36.28 (17)
O1—C9—C10—C15	−86.82 (16)	C16—C8—N1—O2	−91.57 (15)
C8—C9—C10—C15	148.08 (14)	C7—C8—N1—O2	21.31 (19)
C15—C10—C11—C12	−0.8 (3)	C9—C8—N1—O2	148.59 (14)
C9—C10—C11—C12	179.26 (16)	C17—C16—N2—C21	171.62 (12)
C10—C11—C12—C13	0.4 (3)	C8—C16—N2—C21	43.60 (13)
C11—C12—C13—C14	0.2 (3)	C17—C16—N2—C20	−59.32 (17)
C12—C13—C14—C15	−0.4 (3)	C8—C16—N2—C20	172.66 (12)
C13—C14—C15—C10	0.1 (3)	C22—C21—N2—C16	−162.76 (11)
C11—C10—C15—C14	0.5 (2)	C28—C21—N2—C16	83.23 (14)
C9—C10—C15—C14	−179.52 (16)	C7—C21—N2—C16	−38.06 (13)
N1—C8—C16—N2	86.17 (12)	C22—C21—N2—C20	69.69 (16)
C7—C8—C16—N2	−30.59 (13)	C28—C21—N2—C20	−44.32 (17)
C9—C8—C16—N2	−155.88 (11)	C7—C21—N2—C20	−165.61 (12)
N1—C8—C16—C17	−35.65 (17)	C19—C20—N2—C16	57.37 (19)
C7—C8—C16—C17	−152.41 (13)	C19—C20—N2—C21	−178.43 (14)
C9—C8—C16—C17	82.30 (16)	O4—C28—N3—C27	176.25 (15)
N2—C16—C17—C18	58.08 (17)	C21—C28—N3—C27	−0.72 (18)
C8—C16—C17—C18	176.04 (14)	C26—C27—N3—C28	−178.79 (16)
C16—C17—C18—C19	−57.7 (2)	C22—C27—N3—C28	0.01 (18)
C17—C18—C19—C20	56.6 (2)	C6—C1—O1—C9	−40.2 (2)
C18—C19—C20—N2	−55.2 (2)	C2—C1—O1—C9	141.90 (16)
C6—C7—C21—N2	141.84 (12)	C10—C9—O1—C1	−69.57 (16)
C8—C7—C21—N2	17.04 (13)	C8—C9—O1—C1	61.14 (16)
C6—C7—C21—C22	−94.34 (14)		

### Hydrogen-bond geometry (Å, º)

**Table:**

*D*—H···*A*	*D*—H	H···*A*	*D*···*A*	*D*—H···*A*
C16—H16···O4	0.98	2.50	3.1394 (18)	123
N3—H3*A*···O4^i^	0.86	1.97	2.8218 (17)	168

Symmetry code: (i) −*x*+3/2, −*y*+1/2, −*z*+1.
